# Mode of delivery and maternal sexual wellbeing: A longitudinal study

**DOI:** 10.1111/1471-0528.17262

**Published:** 2022-08-03

**Authors:** Florence Z. Martin, Paul Madley‐Dowd, Viktor H. Ahlqvist, Egill Jónsson‐Bachmann, Abigail Fraser, Harriet Forbes

**Affiliations:** ^1^ MRC Integrative Epidemiology Unit Population Health Sciences Bristol Medical School University of Bristol Bristol UK; ^2^ Centre for Academic Mental Health Population Health Sciences Bristol Medical School University of Bristol Bristol UK; ^3^ Department of Global Public Health Karolinska Institutet Stockholm Sweden

**Keywords:** Avon Longitudinal Study of Parents and Children, caesarean section, dyspareunia, vaginal birth

## Abstract

**Objectives:**

To investigate the association between mode of delivery and subsequent maternal sexual wellbeing.

**Design:**

Prospective birth cohort study.

**Setting:**

Avon (in Bristol area), UK.

**Population:**

Participants in the Avon Longitudinal Study of Parents and Children (ALSPAC).

**Methods:**

Mode of delivery was abstracted from obstetric records and sexual wellbeing measures were collected via a self‐report questionnaire. Missing data were imputed using multiple imputation, and ordinal logistic regression models for ordered categorical outcomes were adjusted for the covariates maternal age at delivery, pre‐pregnancy body mass index, diabetes during pregnancy, socio‐economic position, parity, depression and anxiety.

**Main outcome measures:**

Sexual enjoyment and frequency at four time points postpartum (between 33 months and 18 years) and two types of sex‐related pain (pain in the vagina during sex and elsewhere after sex) at 11 years postpartum.

**Results:**

We found no association between mode of delivery and sexual enjoyment (e.g. adjusted odds ratio [OR] 1.11, 95% confidence interval [95% CI] 0.97–1.27 at 33 months) or sexual frequency (OR 0.99, 95% CI 0.88–1.12 at 33 months). Caesarean section was associated with an increased odds of pain in the vagina during sex at 11 years postpartum as compared with vaginal delivery in the adjusted model (OR 1.74, 95% CI 1.46–2.08).

**Conclusions:**

These findings provide no evidence supporting associations between caesarean section and sexual enjoyment or frequency. However, mode of delivery was shown to be associated with dyspareunia, which may not be limited to abdominal scarring.

## 1 INTRODUCTION

Rates of caesarean section have been increasing drastically over the last two decades, rising globally from 7% in 1990 to 19% in 2014^1^; rates in the UK rose from 11% of births in 1990^1^ to 26% in 2015.^2^ There are many reasons as to why this might be, including increasing maternal age, increasing numbers of women who have had a prior caesarean section, and changes in maternal preference.^3^ Among the risks and benefits of caesarean section, it has been suggested that caesarean section maintains sexual wellbeing in both lay and academic channels by reducing the risk of genital damage.^4^ A US study found that one perceived benefit of caesarean section over vaginal delivery is reduced impact on sexual function.^5^ Another UK‐based survey in the mid‐1990s found that one‐third of female obstetricians would choose caesarean section for themselves in part to preserve sexual function, if they had an uncomplicated pregnancy^6^ – a finding which is subject to debate.^7^

The quantitative evidence to support this protective effect of caesarean section on sexual wellbeing is sparse. Short‐term studies have suggested that there is little difference in sexual outcomes at 6 months postpartum when comparing caesarean section with vaginal deliveries.^8^, ^9^, ^10^ A randomised controlled trial where women with a breech‐positioned fetus were assigned to either planned vaginal delivery or caesarean section (Term Breech Trial) found no difference in incidence of sexual problems at 2 years postpartum.^11^ Long‐term evidence is more limited but suggests that caesarean section is associated with increased risk of sexual problems, such as dyspareunia (pain during sex).^12^ It is therefore essential to better understand the impact of mode of delivery on long‐term maternal outcomes including sexual wellbeing and sex‐related pain (referred to together throughout as sex‐related outcomes).

To our knowledge, there are no studies that investigate sexual outcomes across the postpartum period, at multiple timepoints after delivery; however, these outcomes remain a prominent feature in the argument in favour of caesarean section. It is not implausible that anatomical and psychological changes after delivering a baby may affect sexual outcomes that may be different between delivery groups.

The aim of this study was to determine the association between mode of delivery and women’s postpartum sexual outcomes at multiple timepoints up to 18 years after delivery using the Avon Longitudinal Study of Parents and Children (ALSPAC) cohort.

## 2 METHODS

### 2.1 Participants and recruitment

**FIGURE 1:**
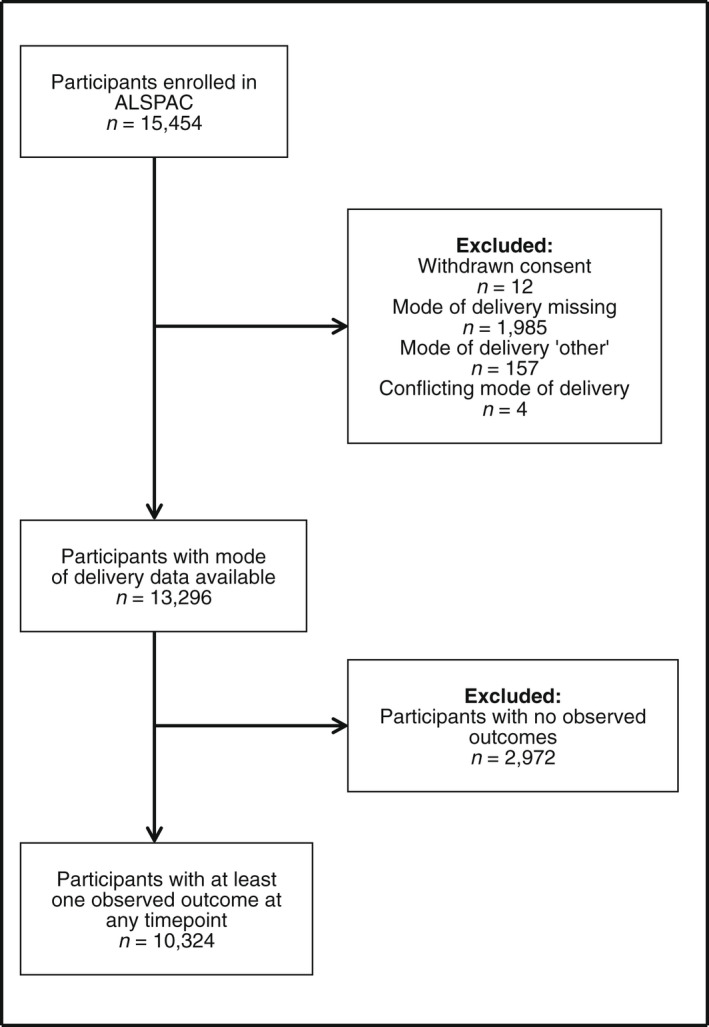
Flow diagram of participants through the study.

ALSPAC is a large prospective cohort of women and their children and partners, recruited between 1990 and 1992 and followed up over the subsequent two decades. The study originally recruited 14 541 pregnant women due to give birth between 1991 and 1992 (referred to as the index pregnancy); a further 913 women who fitted the eligibility criteria have been recruited since. There have been previous publications describing the cohort in full,^13^ as well as online resources via the ALSPAC website for exploring the data.^14^ Please note that the study website contains details of all the data that are available through a fully searchable data dictionary and variable search tool (https://www.bristol.ac.uk/alspac/researchers/our‐data/). Women for whom (index pregnancy) mode of delivery data were available and who had responded to at least one postpartum questionnaire regarding sex‐related outcomes were eligible for inclusion in this study (Figure 1). Ethical approval for this study was obtained from the ALSPAC Ethics and Law Committee, and the local Research Ethics Committee (North Somerset and South Bristol). Informed consent for the use of data collected via questionnaires and clinics was obtained from participants following the recommendations of the ALSPAC Ethics and Law Committee at the time. Patient and public involvement was not employed for this study.

### 2.2 Measures

#### 2.2.1 Mode of delivery

Index pregnancy mode of delivery was abstracted from routine antenatal records by trained research midwives and nurses, where it was recorded as spontaneous vaginal delivery (SVD), caesarean section, assisted breech, breech extraction, forceps, vacuum extraction, or other.^15^ Women with ‘other’ mode of delivery (*n* = 157) or conflicting modes of delivery (*n* = 4) were excluded (Figure 1), to limit exposure misclassification. For the primary exposure, we dichotomised mode of delivery into vaginal delivery (SVD, breech extraction and assisted, forceps and vacuum) (*n* = 9230) and caesarean section (*n* = 1094). As additional analyses, we stratified vaginal delivery into instrumental (forceps and vacuum deliveries) and non‐instrumental (SVD and breech deliveries), and caesarean section into elective and emergency caesarean section (*n* = 1092; for two participants who underwent caesarean section these data were not available). Mode of delivery for previous or subsequent pregnancies was not recorded in ALSPAC and therefore was not included in these analyses.

#### 2.2.2 Sex‐related outcomes

Sex‐related outcomes were measured by self‐reported questionnaires sent out to participants after delivery. Sexual enjoyment and frequency were measured multiple times; we chose four timepoints *a priori*, 33 months, 5, 12 and 18 years postpartum, to investigate both outcomes at different times, moving further away temporally from the index birth. Questions relating to sexual enjoyment and frequency were in questionnaires asking about general health and lifestyle.^16^ Pain, both in the vagina during sex and elsewhere after sex, was measured once at 11 years postpartum. These questions were included in a questionnaire about health events and were chosen based on relevance to the outcomes of interest.^16^ These questionnaires were not validated for specific outcomes.

Women were asked if they enjoyed sexual intercourse, giving possible responses ‘yes, very much’, ‘yes, somewhat’, ‘no, not a lot’, ‘no, not at all’ and ‘no sex at the moment’.

Sexual frequency was assessed with questions that gave possible responses ‘not at all’, ‘less than once a month’, ‘1–3 times a month’, ‘about once a week’, ‘2–4 times a week’ and ‘5 or more times a week’.

Sex‐related pain was assessed by two questions: presence of pain in the vagina during sex and presence of pain elsewhere after sex. Pain in the vagina during sex was assessed by a question that gave the following possible responses ‘not at all’, ‘a little’, ‘moderate’ and ‘a lot’. The question investigating pain elsewhere after sex gave possible responses ‘don’t have sex’, ‘never’, ‘occasionally’, ‘often’ and ‘always’.

For women who responded ‘no sex at the moment’ or ‘don’t have sex’ to sexual enjoyment and pain elsewhere after sex, respectively, we were unable to ascertain the reason as to why women were not having sex. It was plausible that they were not having sex because of low enjoyment or increased pain. Therefore values were recoded as missing and imputed for the main analysis (for sexual enjoyment *n* = 634, *n* = 572, *n* = 583 and *n* = 564 recoded at 33 months, 5, 12 and 18 years, respectively, and pain elsewhere after sex *n* = 173 recoded at 11 years), and then examined in a sensitivity analysis.

### 2.3 Statistical analysis

Confounders were identified *a priori* from published literature as factors that may have affected both mode of delivery and sexual outcomes: maternal age,^17^, ^18^ body mass index (BMI),^19^, ^20^ diabetes,^21^, ^22^ parity,^23^ socio‐economic position,and mental health problems (depression and anxiety)^24^, ^25^ (data collection detailed in Appendix S1).

We summarised the extent of missingness for all variables in the analysis, in order to determine whether observed data were associated with being a complete case,^26^, ^27^ as well as the proportion of missing outcome data for participants who did complete compared with those who did not complete each questionnaire (Tables S2–S8). We also determined the proportion of women who completed the questionnaire but did not answer the sex‐related questions and compared this with other questions in the same questionnaire, to investigate the missingness mechanism in our outcomes. Given the majority of the outcome and covariate data were deemed to be Missing at Random and availability of good auxiliaries in ALSPAC, we used multiple imputation (MI) to address missing covariables and outcome data^28^ (detailed in Appendix S1).

We used ordinal logistic regression for all outcomes, to estimate the effect of the exposure (mode of delivery) on maternal sexual outcomes, with the odds ratio (OR) indicating the odds of reporting a one‐level higher category of enjoyment, frequency or pain in women who underwent caesarean section compared with women who had a vaginal delivery. In other words, an OR above one for enjoyment/frequency denotes a positive outcome (more enjoyable/frequent), whereas an OR above one for sex‐related pain denotes a negative outcome (more pain), for women who gave birth via caesarean section compared with those who delivered vaginally. The Brant test was used to assess whether the proportional odds assumption was violated in each model (see Appendix S1 in Table S1).

We performed two additional analyses comparing sex‐related outcomes between instrumental and non‐instrumental vaginal deliveries, as well as emergency and elective caesarean section (separately) with vaginal delivery.

### 2.4 Sensitivity analyses

Given the potential for bias introduced by a small number of participants who had completed questionnaires but selectively missed the sex‐related questions (thus potentially Missing Not At Random [MNAR]), we repeated analyses under a ‘worst‐case scenario’, in which they were assigned the lowest level of enjoyment/frequency (for sexual enjoyment *n* = 274, *n* = 385, *n* = 443 and *n* = 292, and for sexual frequency *n* = 173, *n* = 295, *n* = 314 and *n* = 218 recoded at 33 months, 5, 12 and 18 years, respectively). Similarly for sex‐related pain, those who may be MNAR (returned the questionnaire but missed the sex‐related pain questions) were recoded as the highest category for each question (for pain in the vagina *n* = 410 and pain elsewhere *n* = 426).

The participants who responded ‘no sex at the moment’ or ‘don’t have sex’ (who were recoded to missing and imputed for the primary analysis) were set to ‘no, not at all’ (for sexual enjoyment) and ‘yes, a lot’ (for pain elsewhere after sex), the most extreme values for each variable, in the ‘worst‐case scenario’ sensitivity analysis.

We repeated our MI analysis having recoded MNAR and ‘no sex at the moment’ participants to ‘worst‐case scenario’ categories, to understand how robust the results of the primary analysis were to the decision of imputing these participants in the primary analysis. We also repeated our MI analysis with the additional adjustment of self‐reported general health in order further to account for differential comorbidity between exposure groups.

We also performed a complete case analysis (CCA) for caesarean section compared with vaginal delivery, and additionally performed this analysis in nulliparous women, in an attempt to mitigate confounding by previous births not recorded in ALSPAC. All analyses were completed in STATA (version 17.1) (StataCorp).

## 3 RESULTS

### 3.1 Study sample

Participation rate across timepoints declined in ALSPAC. Of the total sample, sexual enjoyment responses decreased from 8673 (56%) at 33 months to 3275 (21%) at 18 years postpartum. Sexual frequency responses decreased from 9439 (61%) at 33 months to 3945 (26%) at 18 years postpartum.

Figure 1 shows the study flow and Table 1 reports the characteristics of participants in the imputed sample (*n* = 10 324), by either vaginal delivery or caesarean section. Women who delivered via caesarean section (11% of the sample) tended to be older, have a higher mean body mass index (BMI; 24.2 in caesarean section versus 22.8 for vaginal delivery) and were more likely to be nulliparous at the time of pregnancy with their index child (54% for caesarean section versus 44% for vaginal delivery).

**TABLE 1:** Characteristics of participants with complete exposure data (either caesarean section or vaginal delivery) and at least one observed outcome (*n* = 10 324)

	Caesarean section, *n* (%)	Vaginal delivery^a^, *n* (%)
Number of observations	1094 (100)	9230 (100)
BMI^b^ (pre‐pregnancy)		
Mean BMI, kg/m^2^ (SD)	24.2 (4.7)	22.8 (3.7)
Missing	123 (11.2)	969 (10.5)
Maternal age at delivery		
Mean age, years (SD)	29.5 (4.8)	28.4 (4.7)
Missing	0 (0)	0 (0)
Diabetes during pregnancy		
Prevalent or gestational	37 (3.4)	46 (0.5)
Missing	0 (0)	0 (0)
Anxiety (18 weeks’ gestation)		
CCEI^c^ Score ≥8	167 (17.2)	1266 (15.5)
Missing	124 (11.3)	1050 (11.4)
Depression (18 weeks’ gestation)		
EPDS^d^ Score ≥ 13	132 (13.5)	1023 (12.3)
Missing	118 (10.8)	918 (9.9)
Parity (18 weeks’ gestation)		
Multiparous	489 (46.4)	4948 (55.7)
Missing	41 (3.8)	351 (3.8)
Maternal education (32 weeks’ gestation)		
University degree	139 (13.4)	1246 (14.1)
Missing	55 (5.0)	364 (3.9)

Abbreviation: SD, Standard deviation.

^a^ Vaginal delivery: spontaneous vaginal delivery, breech deliveries, forceps delivery & vacuum delivery.

^b^ Body mass index.

^c^ Crown Crisp Experimental Index (CCEI) for anxiety: threshold (≥8 for probable anxiety) as determined by Glover et al.^41^

^d^ Edinburgh Postnatal Depression Scale (EPDS) for depression: threshold (≥13 for probable depression of varying degrees of severity) as determined by Cox et al.^42^ of the 10‐item EPDS.

**FIGURE 2:**
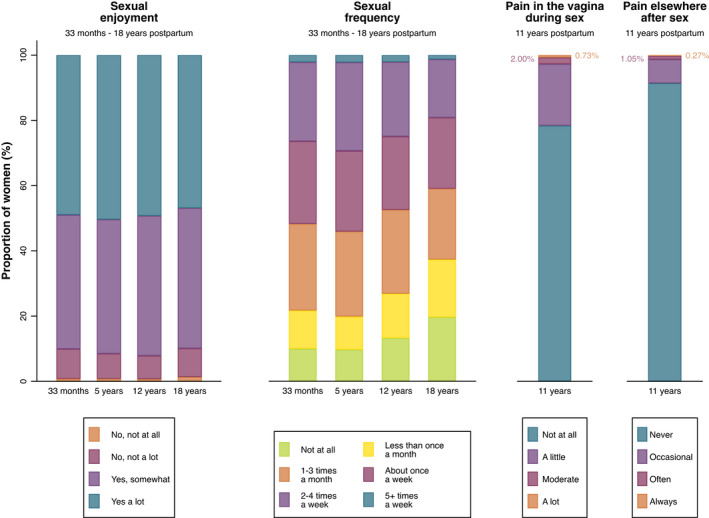
Proportion of participants who responded to each question, pertaining to each outcome of sexual enjoyment, sexual frequency and sex‐related pain at each timepoint.

Distributions of sexual enjoyment and frequency are presented in Figure 2, and remained stable over time; for sex‐related pain, the majority of respondents reported no pain, either in the vagina during sex and elsewhere after sex (stratified by mode of delivery Figures S1 and S2).

### 3.2 Primary analysis

**FIGURE 3:**
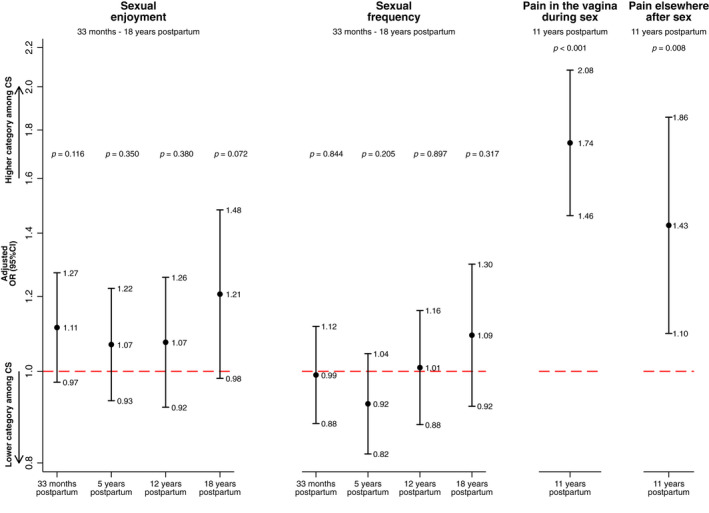
Adjusted odds ratio of being at a higher level of the ranked outcome, comparing caesarean section with vaginal delivery for each outcome: sexual enjoyment, sexual frequency and sex‐related pain at each timepoint (*n* = 10 324). Models adjusted for: maternal age at delivery, maternal BMI (12 weeks' gestation), maternal diabetes (12 weeks' gestation), maternal anxiety (18 weeks' gestation), maternal depression (18 weeks' gestation), parity (18 weeks' gestation) and maternal educational attainment (32 weeks' gestation).

There was no association between mode of delivery and sexual enjoyment in the adjusted models at any timepoint (adjusted odds ratio [aOR] 1.11, 95% confidence interval [CI] 0.97–1.27 at 33 months and aOR 1.21, 95% CI 0.98–1.48 at 18 years postpartum) (Figure 3, Table S9).

Mode of delivery was not associated with sexual frequency (aOR, 0.99; 95% CI 0.88–1.12 at 33 months and aOR 1.09; 95% CI 0.92–1.30 at 18 years postpartum) (Figure 3, Table S9).

Sexual frequency at 5 years violated the proportional odds assumption (Table S16). We believe this is likely to be chance, as the finding is not consistent for other comparisons made by the model, e.g. about once a week compared with not at all, and should not be over‐interpreted.

Women who delivered via caesarean section were more likely to report pain in the vagina during sex and pain elsewhere after sex than were women who delivered vaginally (aOR 1.74, 95% CI 1.46–2.08 and aOR 1.43, 95% CI 1.10–1.86, respectively) (Figure 3, Table S9).

### 3.3 Additional analyses

Associations between instrumental vaginal delivery (versus non‐instrumental vaginal delivery) and sexual enjoyment, sexual frequency and sex‐related pain are presented in Table S10. There was no evidence of associations between type of vaginal delivery and outcomes in adjusted models. Where we compared emergency and elective caesarean section with vaginal delivery separately, findings were consistent with the primary analysis (Table S11), where both types of caesarean section were associated with increased sex‐related pain.

### 3.4 Sensitivity analyses

In the ‘worst‐case scenario’ analysis, results were virtually unchanged for sexual enjoyment, sexual frequency and pain in the vagina during sex. For pain elsewhere after sex, the point estimate attenuated to the null compared with the main analysis, weakening the evidence for an association for this outcome (Table S12). After additionally adjusting for general health, to better account for morbidity associated with mode of delivery and subsequent sexual wellbeing, our conclusions did not change and pain in the vagina remained higher among those who delivered via caesarean section (Table S13).

The CCA sensitivity analysis showed very similar results to the primary imputed analysis (Table S14). When comparing caesarean section and vaginal delivery in nulliparous women, the same results for all outcomes at all timepoints were observed, except pain elsewhere after sex, which attenuated towards the null compared with the primary analysis (Table S15). We did not perform this sensitivity analysis in the imputed dataset as we had imputed parity, and restricting to nulliparous prior to imputation led to perfect prediction in the imputation model. Given that the results from the CCA were comparable with the imputed primary analysis, we deemed it acceptable to run this sensitivity analysis in the complete case cohort.

## 4 DISCUSSION

### 4.1 Main findings

We found no evidence of an association between mode of delivery and sexual enjoyment or frequency in the early or distal postpartum periods. However, we did observe an increase in odds of pain in the vagina during sex at 11 years postpartum for women who delivered via caesarean section as compared with those who delivered vaginally. The findings from the primary analysis for pain elsewhere after sex were not robust to sensitivity analyses, and we are therefore more cautious when interpreting this outcome.

### 4.2 Strengths and limitations

The strengths of this study include long‐term follow‐up with linkage to clinical records and repeat measures of sexual enjoyment and frequency. To our knowledge there are no studies that present sexual enjoyment and frequency outcomes alongside sex‐related pain, comparing modes of delivery at multiple timepoints postpartum. Stratification of caesarean section is one of the main strengths of this study, which is lacking in the current literature.

The absence of longitudinal data on dyspareunia that predates pregnancy in this study is a limitation, meaning we could not assess confounding by indication (sex‐related pain potentially predicting mode of delivery). Secondly, we did not have women’s complete obstetric history, only data on the mode of delivery of the index pregnancy in ALSPAC; this a major issue that needs future studies including every delivery for each participant. However, when the complete case analysis was performed among those who were nulliparous comparing caesarean section and vaginal delivery, we found the same increased risk of sex‐related pain among women who delivered their first child via caesarean section. The questions regarding sexual wellbeing were not validated, such as the Female Sexual Function Index (FSFI) (measuring six dimensions of sexual function including arousal, satisfaction and pain).^29^ Additionally, the inability to ascertain where participants were experiencing pain when reporting ‘pain elsewhere after sex’ limits our ability to draw conclusions about this particular outcome. Finally, the inability further to investigate the role of episiotomy and perineal tear in each delivery group for each sexual outcome was a limitation, as both have been identified in the literature to be associated with postpartum dyspareunia.^30^, ^31^

### 4.3 Interpretation

To our knowledge, no studies have investigated the associations between mode of delivery and sexual enjoyment beyond the early postpartum period. However, our findings for sexual enjoyment are consistent with studies that carried out analyses investigating similar outcomes in the first 2 years following childbirth. One Iranian study that followed up participants at 3–6 months postpartum found that FSFI scores did not differ by mode of delivery.^8^ Another Iranian study found no difference in FSFI scores between women who gave birth vaginally and women who gave birth via an elective caesarean section 24 months postpartum.^32^

Similarly, this is the first study investigating the association of mode of delivery with sexual frequency. Although it may be less important for wellbeing than sexual enjoyment or sex‐related pain, it is an important measure to observe alongside other sexual outcomes.

Dyspareunia is more frequently explored when comparing mode of delivery groups. An Australian study concluded that women who gave birth via emergency caesarean section were more likely to experience dyspareunia by 18 months postpartum than were women who gave birth via spontaneous vaginal delivery (aOR 2.41, 95% CI 1.40–4.00); however, large standard errors reduced the strength of evidence from that study.^33^ A large Danish cohort study found that women who gave birth exclusively via caesarean section were more likely to experience sexual problems, including both deep (aOR 1.25, 95% CI 1.08–1.45) and entry dyspareunia (aOR 2.76, 95% CI 2.36–3.24) up to 16 years postpartum,^12^ compared with those who delivered exclusively via SVD. In contrast to our findings, a randomised controlled trial found no difference in sex‐related pain between planned caesarean section and planned vaginal delivery groups in breech pregnancies; however, this outcome was not adequately analysed.^11^ Our study was performed in a different population to the Term Breech Trial, potentially reducing comparability between the two. Also, the intention‐to‐treat effect may have attenuated to the null, as only 55% of the women with planned vaginal birth went on to deliver vaginally. In spite of these explanations, it is plausible that our study suffered from residual confounding, circumvented by the Term Breech Trial by randomisation.

If our finding is causal, a potential explanation for abdominal pain associated with sex could be related to the caesarean scar or uterine scarring (also known as a niche).^34^ A systematic review suggested the prevalence of niches was 56–84% in women who had undergone a caesarean section,^35^ and a recent focus group identified sexual activity as a key issue for women with niches, including reduced frequency of sexual intercourse and dyspareunia.^36^ Alternatively, it is also possible that dyspareunia could be a predictor for having difficulty giving birth vaginally, potentially leading to caesarean section, implying that confounding by indication may be contributing to this finding. The association between dyspareunia and subsequent caesarean section as used to determine whether our analysis is confounded by indication, is an important question yet to be answered; however, one study found that vaginismus and localised provoked vestibulodynia, two of the most common causes of dyspareunia,^37^, ^38^ are associated with caesarean section,^39^ supporting our suggestion of confounding by indication.

Dyspareunia is a complex phenotype with many suggested etiologies that differ between women who experience painful sex.^40^ It is plausible that a traumatic birth resulting in an emergency caesarean section, along with the psychosocial pressures of giving birth ‘naturally’ as opposed to operatively, could play a role in sexual outcomes postpartum in some cases.

Although these findings will not affect obstetric decision‐making during labour, they may aid in destigmatizing sex‐related pain experienced by women having delivered via caesarean section and open the dialogue with their clinician regarding dyspareunia after delivery. For women considering a planned caesarean section in an uncomplicated pregnancy, evidence suggesting that caesarean section may not protect against sexual dysfunction may help inform their decision‐making in the antenatal period.

Overall, these findings will inform the debate surrounding the effect of caesarean section on maternal sexual wellbeing postpartum, which is particularly relevant in the context of rising rates of both emergency and planned caesarean sections.

Sex‐related pain must be considered across the life course in future studies. To tease apart the potential confounding by indication, where dyspareunia may be a predictor of caesarean section, longitudinal data on sex‐related pain need to be collected both before and after parturition. Taking a life‐course approach to investigating the association between mode of delivery and sex‐related pain in the future will elucidate whether caesarean section could be causing sex‐related pain or whether sexual dysfunction, resulting in pain, is influencing mode of delivery.

## 5 CONCLUSION

This study suggests that mode of delivery is not associated with sexual enjoyment or frequency postpartum. Contrary to current qualitative perceptions, our data suggest that caesarean section was associated with increased odds of experiencing pain in the vagina during sex compared with vaginal delivery, consistent with other quantitative studies. It has highlighted the need for more research into caesarean section and its long‐term maternal wellbeing outcomes, including clarifying the direction of a possible effect between caesarean section and dyspareunia.

## AUTHOR CONTRIBUTIONs

HF and AF conceived the study, FZM helped design the study with them for an MSc dissertation project. FZM analysed the data and drafted the manuscript. PMD and AF provided additional support in the design of the missing data approach. PMD, VHA, EJB, HF and AF aided FZM in interpretation of the data and provided several rounds of revisions to the manuscript. HF and AF are joint last authors on this manuscript and FZM, HF and AF are the guarantors.

## FUNDING INFORMATION

The UK Medical Research Council, the Wellcome Trust (Grant ref: 217065/Z/19/Z) and the University of Bristol provide core support for ALSPAC. This publication is the work of authors, FZM, HF and AF will act as guarantors for the contents of this paper. This research was funded in whole, or in part, by the Wellcome Trust (Grant number: 218495/Z/19/Z). For the purpose of Open Access, the author has applied a CC BY public copyright licence to any Author Accepted Manuscript version arising from this submission. A comprehensive list of grants funding is available on the ALSPAC website (http://www.bristol.ac.uk/alspac/external/documents/grant‐acknowledgements.pdf). The funders of this project had no role in the design or conduct (including analysis or interpretation) of this study, or in the decision to submit this manuscript for publication.

## CONFLICT OF INTERESTS

None declared. Completed disclosure of interest forms are available upon request from the corresponding author.

## ETHICS STATEMENT

Approval for this study was obtained from the ALSPAC Ethics and Law Committee, and the local Research Ethics Committee (North Somerset and South Bristol). Informed consent for the use of data collected via questionnaires and clinics was obtained from participants following the recommendations of the ALSPAC Ethics and Law Committee at the time.

## Supporting information


Appendix S1

Figure S1

Figure S2

Data S1

Data S2

Data S3

Data S4

Data S5

Data S6

## Data Availability

The data that support the findings of this study are available from ALSPAC Executive. Restrictions apply to the availability of these data, which were used under license for this study. Data are available at http://www.bristol.ac.uk/alspac/researchers/access/ with the permission of ALSPAC Executive.
