# Supplementary material for: Mode of delivery and maternal sexual wellbeing: A longitudinal study
Source: BJOG. 2022 Aug 3;129(12):2010–8. doi: 10.1111/1471-0528.17262 (PMC9804306; doi:10.1111/1471-0528.17262)
Supplement: Supplementary file 1 — Appendix S1 [file BJO-129-2010-s009.docx]

**Supporting information**

Mode of delivery and maternal sexual wellbeing: a longitudinal investigation of a UK-based cohort

**A – Methods**

**A.1 – Confounders**

Age, pre-pregnancy BMI, and parity were collected over the course of pregnancy at various timepoints, via questionnaire. Maternal depression and anxiety during pregnancy were measured using the Edinburgh Postnatal Depression Scale^2^ (EPDS) score and Crown Crisp Experimental Index^1^ (CCEI) score, respectively. Parity, EPDS score, and CCEI score were binarised for the purpose of the demographic comparison in Table 1, however were collected as quasi continuous variables in ALSPAC.

Diabetes during pregnancy, including both prevalent and gestational diabetes, was abstracted from the obstetric records, similarly to mode of delivery.

Socioeconomic position (SEP) has been shown to be associated with differing rates of caesarean section in multiple cohorts;^3^ although direct associations between SEP and the sexual outcomes described here haven’t, to our knowledge, been examined in the literature, we deemed it appropriate to include SEP as a confounder. We used highest maternal educational attainment as a proxy measure of SEP.

General health was obtained at 32 weeks’ gestation to better capture chronic health problems that may be differential between delivery groups. Participants were asked “how would you describe your health in the last two weeks?” to which they could respond “always fit and well”, “usually fit and well”, “often unwell”, and “always unwell”. General health was imputed in an additional dataset and adjusted for in a sensitivity analysis (Table S12).

**A.2 – Assessment of missing data**

In order to fully justify our choice of methods to appropriately deal with missing data, we were guided by the Lee *et al*. framework for treating and reporting missing data.^4^ Within this framework exists a flow diagram that asks authors first to consider whether a complete case analysis of their data may be biased.^4^ We argued that although there was evidence to suggest that a complete case analysis may not be biased, due to the observed outcomes not appearing to be associated with being a complete case, we could not rule out the possibility that the unobserved outcomes may have influenced missingness of outcomes. For this reason, and that multiple imputation may have increased efficiency (due to the availability of adequate auxiliaries for our covariates and outcomes in ALSPAC), we deemed multiple imputation as a justifiable approach to handle the missing data in this study.

In order to determine whether there was evidence to suggest that the unobserved outcomes for each timepoint were missing at random (MAR) or missing not at random (MNAR), we explored the missing outcome data in more detail. First, we ascertained how many participants had missing data at each timepoint for each outcome; out of these we calculated the proportion of those with missing data who had returned the questionnaire and those who had not. For those who did not return the questionnaire, we believed data was not missing dependent on the unobserved response to the sex-related question and was therefore MAR given sociodemographic information which were accounted for in the analysis model. For those who had returned the questionnaire but not answered the sex-related question used to define our outcome, we believed that this was evidence for MNAR outcome data, in other words the outcome (the answer to the question) may have influenced the participant to not answer the question (resulting in a missing value).

In order to explore the potential MNAR pattern further, we looked at rates of missingness in other questions in each questionnaire. This was to understand whether questionnaire fatigue or chance was playing a role in selective answering for some participants. For each questionnaire, we chose the question immediately prior to our sex-related questionnaire and a randomly chosen subsequent question. Within those who returned the questionnaire, we calculated the proportion of people who did not answer the prior, sex-related, and subsequent questions. If there was a comparable amount of missing data in the other questions, this would provide evidence that those who had returned the questionnaire but not answered those questions was down to chance or questionnaire fatigue. If there was more missing data in the sex-related questions among those who had returned the questionnaire, this would provide more evidence for the outcome being partially MNAR.

For the multiple imputation, we used auxiliaries for covariates, as well as observed outcomes as auxiliaries for unobserved outcomes, hence the rationale to include those with complete exposure data and at least one complete outcome. In order to confirm that using observed outcomes as auxiliaries was appropriate, we performed simple unadjusted ordinal logistic regressions of each outcome at each timepoint on all other outcomes. Although crude, this provided confirmation that each outcome was associated with the other outcomes at different timepoints and reassurance that they made good auxiliary variables for the multiple imputation model.

**A.3 – Multiple imputation model**

All variables in the analysis model, and all auxiliaries were included in the imputation model. Auxiliaries included absolute weight gain during pregnancy and anxiety and depression at 33 months postpartum; sexual outcomes acted as auxiliaries for each other. Sexual outcomes were imputed using ordinal logistic regression. Predictive mean matching, selecting from the 10 nearest neighbours, was used to impute parity, anxiety, and depression. Other variables were imputed using either multinomial logistic or linear regression. MI was conducted using the “mi impute chained” command in Stata, imputing 100 datatsets.

**A.4 – Brant tests**

**Table S1.** In order to test the proportional odds assumption to uphold the use of ordinal logistic regression, ordered logit estimates and the Brant test were used, where the null hypothesis states that there is no difference in coefficients between categories thus the proportional odds assumption isn’t violated.

| **Outcome** | **Ordered logit estimate likelihood ratio test of odds across response categories** | **Brant test of parallel regression assumption** |
| --- | --- | --- |
| Sexual enjoyment at 33 months postpartum | 0.259 | 0.348 |
| Sexual enjoyment at 5 years postpartum | 0.623 | 0.628 |
| Sexual enjoyment at 12 years postpartum | 0.369 | 0.496 |
| Sexual enjoyment at 18 years postpartum | 0.247 | 0.290 |
| Sexual frequency at 33 months postpartum | 0.126 | 0.149 |
| Sexual frequency at 5 years postpartum | 0.012 | 0.020 |
| Sexual frequency at 12 years postpartum | 0.217 | 0.251 |
| Sexual frequency at 18 years postpartum | 0.418 | 0.400 |
| Pain in the vagina during sex at 11 years postpartum | 0.912 | 0.915 |
| Pain elsewhere after sex at 11 years postpartum | 0.893 | 0.909 |

None of the models violated the proportional odds assumption except sexual frequency at 5 years postpartum. In this scenario, a set of generalized ordered logit estimates may be generated (using ‘gologit2’ which relaxes the proportional odds assumption and provides an estimate for each category). Due to the difficulty of using the ‘gologit2’ model with imputed data, ‘ologit’ was used sexual frequency at 5 years postpartum in the primary analysis and additional analyses, then checked in the complete case cohort against the generalized model (Table S15).

**B – Results**

**B.1 – Assessment of missing data**

**Table S2.** Summary of the variables in the analysis model for sexual enjoyment at each timepoint, including the amount of data available for each variable in both the full dataset (after exclusions) and restricted to those with observed outcome data.

| **Characteristic** | **Categories** | **Available data**  **(*n*=13,296)**  ***n* (%)** | **Available data by category^1^**  ***n* (% of available data)** | **Complete records 33 months**  **(*n*=7,190)**  ***n* (%)** | **Complete records 5 years**  **(*n*=6,609)**  ***n* (%)** | **Complete records 12 years**  **(*n*=4,969)**  ***n* (%)** | **Complete records 18 years**  **(*n*=3,111)**  ***n* (%)** |
| --- | --- | --- | --- | --- | --- | --- | --- |
| Mode of delivery | Vaginal delivery | 13,296 (100) | 11,845 (89) | 6,454 (90) | 5,934 (90) | 4,457 (90) | 2,796 (90) |
|  | Caesarean section |  | 1,451 (11) | 736 (10) | 675 (10) | 512 (10) | 315 (10) |
| Body mass index (BMI) | Underweight | 11,024 (83) | 553 (5) | 319 (4) | 292 (4) | 206 (4) | 126 (4) |
|  | Normal |  | 8,186 (74) | 5,368 (75) | 4,950 (75) | 3,792 (76) | 2,397 (77) |
|  | Overweight |  | 1,670 (15) | 1,105 (15) | 1,017 (15) | 721 (15) | 450 (14) |
|  | Obese |  | 615 (6) | 398 (6) | 350 (5) | 250 (5) | 138 (4) |
| Age | Under 25 | 13,296 (100) | 3,211 (24) | 1,162 (16) | 1,027 (16) | 675 (14) | 368 (12) |
|  | 25 and over |  | 10,085 (76) | 6,028 (84) | 5,582 (84) | 4,294 (86) | 2,743 (88) |
| Diabetes during pregnancy | No | 13,296  (100) | 13,190 (99) | 7,136 (99) | 6,560 (99) | 4,931 (99) | 3,087 (99) |
|  | Yes |  | 106 (1) | 54 (1) | 49 (1) | 38 (1) | 24 (1) |
| Maternal education | A levels or less | 11,889 (89) | 10,374 (87) | 6,120 (85) | 5,598 (85) | 4,110 (83) | 2,476 (80) |
|  | University degree |  | 1,515 (13) | 1,070 (15) | 1,011 (15) | 859 (17) | 635 (20) |
| Anxiety | CCEI^2^ Score <8 | 11,241 (85) | 9,373 (83) | 6,138 (85) | 5,649 (85) | 4,292 (86) | 2,711 (87) |
|  | CCEI^2^ Score ≥8 |  | 1,868 (17) | 1,052 (15) | 960 (15) | 677 (14) | 400 (13) |
| Depression | EPDS^3^ Score <13 | 11,431 (86) | 9,841 (86) | 6,379 (89) | 5,852 (89) | 4,448 (90) | 2,804 (90) |
|  | EPDS^3^ Score ≥13 |  | 1,590 (14) | 811 (11) | 757 (11) | 521 (10) | 307 (10) |
| Parity | Nulliparous | 12,327 (93) | 5,485 (45) | 3,229 (45) | 2,990 (45) | 2,351 (47) | 1,478 (47) |
|  | Multiparous |  | 6,842 (55) | 3,961 (55) | 3,619 (55) | 2,618 (53) | 1,633 (53) |
| Sexual enjoyment at 33 months postpartum | No sex at the moment | 9,037 (68) | 634 (7) | 493 (7) | - | - | - |
|  | Not at all |  | 65 (1) | 51 (1) | - | - | - |
|  | Not a lot |  | 766 (8) | 614 (9) | - | - | - |
|  | Somewhat |  | 3,457 (38) | 2,775 (39) | - | - | - |
|  | Very much |  | 4,115 (46) | 3,257 (45) | - | - | - |
| Sexual enjoyment at 5 years postpartum | No sex at the moment | 8,278 (62) | 572 (7) | - | 440 (7) | - | - |
|  | Not at all |  | 60 (1) | - | 50 (1) | - | - |
|  | Not a lot |  | 595 (7) | - | 477 (7) | - | - |
|  | Somewhat |  | 3,169 (38) | - | 2,560 (39) | - | - |
|  | Very much |  | 3,882 (47) | - | 3,082 (47) | - | - |
| Sexual enjoyment at 12 years postpartum | No sex at the moment | 6,078 (46) | 583 (10) | - | - | 460 (9) | - |
|  | Not at all |  | 38 (1) | - | - | 30 (1) | - |
|  | Not a lot |  | 398 (7) | - | - | 320 (6) | - |
|  | Somewhat |  | 2,354 (39) | - | - | 1,966 (40) | - |
|  | Very much |  | 2,705 (45) | - | - | 2,193 (44) | - |
| Sexual enjoyment at 18 years postpartum | No sex at the moment | 3,714 (28) | 564 (15) | - | - | - | 461 (15) |
|  | Not at all |  | 42 (1) | - | - | - | 36 (1) |
|  | Not a lot |  | 279 (7) | - | - | - | 240 (8) |
|  | Somewhat |  | 1,359 (37) | - | - | - | 1,164 (37) |
|  | Very much |  | 1,470 (40) | - | - | - | 1,210 (39) |

^1^ By category out of total number of participants with data for each characteristic

^2^ Crown Crisp Experimental Index (CCEI) for anxiety: threshold (≥8 for probable anxiety) as determined by authors Glover *et al*. (2004) ^1^

^3^ Edinburgh Postnatal Depression Scale (EPDS) for depression: threshold (≥13 for probable depression of varying degrees of severity) as determined by authors Cox *et al*. (1987) ^2^ of the 10-item EPDS

**Table S3.** Summary of the variables in the analysis model for sexual frequency at each timepoint, including the amount of data available for each variable in both the full dataset (after exclusions) and restricted to those with observed outcome data.

| **Characteristic** | **Categories** | **Available data**  **(*n*=13,296)**  ***n* (%)** | **Available data by category^a^**  ***n* (% of available data)** | **Complete records 33 months**  **(*n*=7,254)**  ***n* (%)** | **Complete records 5 years**  **(*n*=6,675)**  ***n* (%)** | **Complete records 12 years**  **(*n*=5,059)**  ***n* (%)** | **Complete records 18 years**  **(*n*=3,169)**  ***n* (%)** |
| --- | --- | --- | --- | --- | --- | --- | --- |
| Mode of delivery | Vaginal delivery | 13,296 (100) | 11,845 (89) | 6,514 (90) | 5,991 (90) | 4,545 (90) | 2,853 (90) |
|  | Caesarean section |  | 1,451 (11) | 740 (90) | 684 (10) | 514 (10) | 316 (10) |
| Body mass index (BMI) | Underweight | 11,024 (83) | 553 (5) | 327 (5) | 296 (4) | 212 (4) | 126 (4) |
|  | Normal |  | 8,186 (74) | 5,417 (75) | 5,000 (75) | 3,856 (76) | 2,443 (77) |
|  | Overweight |  | 1,670 (15) | 1,112 (15) | 1,023 (15) | 738 (15) | 458 (14) |
|  | Obese |  | 615 (6) | 398 (5) | 356 (5) | 253 (5) | 142 (4) |
| Age | Under 25 | 13,296 (100) | 3,211 (24) | 1,179 (16) | 1,036 (16) | 687 (14) | 372 (12) |
|  | 25 and over |  | 10,085 (76) | 6,075 (84) | 5,639 (84) | 4,372 (86) | 2,797 (88) |
| Diabetes during pregnancy | No | 13,296  (100) | 13,190 (99) | 7,199 (99) | 6,625 (99) | 5,021 (99) | 3,144 (99) |
|  | Yes |  | 106 (1) | 55 (1) | 50 (1) | 38 (1) | 25 (1) |
| Educational attainment | A levels or less | 11,889 (89) | 10,374 (87) | 6,173 (85) | 5,654 (85) | 4,185 (83) | 2,522 (80) |
|  | University degree |  | 1,515 (13) | 1,081 (15) | 1,021 (15) | 874 (17) | 647 (20) |
| Anxiety | CCEI^b^ Score <8 | 11,241 (85) | 9,373 (83) | 6,184 (85) | 5,699 (85) | 4,366 (86) | 2,759 (87) |
|  | CCEI^b^ Score ≥8 |  | 1,868 (17) | 1,070 (15) | 976 (15) | 693 (14) | 410 (13) |
| Depression | EPDS^c^ Score <13 | 11,431 (86) | 9,841 (86) | 6,427 (89) | 5,903 (88) | 4,525 (89) | 2,853 (90) |
|  | EPDS^c^ Score ≥13 |  | 1,590 (14) | 827 (11) | 772 (12) | 534 (11) | 316 (10) |
| Parity | Nulliparous | 12,327 (93) | 5,485 (45) | 3,264 (45) | 3,020 (45) | 2,388 (47) | 1,500 (47) |
|  | Multiparous |  | 6,842 (55) | 3,990 (55) | 3,655 (55) | 2,671 (53) | 1,669 (53) |
| Sexual frequency at 33 months postpartum | Not at all | 9,138 (69) | 903 (10) | 703 (10) | - | - | - |
|  | < once a month |  | 1,071 (12) | 854 (12) | - | - | - |
|  | 1-3 times a month |  | 2,429 (27) | 1,978 (27) | - | - | - |
|  | About once a week |  | 2,319 (25) | 1,843 (25) | - | - | - |
|  | 2-4 times a week |  | 2,218 (24) | 1,727 (24) | - | - | - |
|  | >5 a week |  | 198 (2) | 149 (2) | - | - | - |
| Sexual frequency at 5 years postpartum | Not at all | 8,368 (63) | 810 (10) | - | 620 (9) | - | - |
|  | < once a month |  | 853 (10) | - | 686 (10) | - | - |
|  | 1-3 times a month |  | 2,179 (26) | - | 1,765 (26) | - | - |
|  | About once a week |  | 2,054 (25) | - | 1,651 (25) | - | - |
|  | 2-4 times a week |  | 2,285 (27) | - | 1,811 (27) | - | - |
|  | >5 a week |  | 187 (2) | - | 142 (2) | - | - |
| Sexual frequency at 12 years postpartum | Not at all | 6,207 (47) | 819 (13) | - | - | 626 (12) | - |
|  | < once a month |  | 845 (14) | - | - | 695 (14) | - |
|  | 1-3 times a month |  | 1,597 (26) | - | - | 1,322 (26) | - |
|  | About once a week |  | 1,389 (22) | - | - | 1,152 (23) | - |
|  | 2-4 times a week |  | 1,425 (23) | - | - | 1,159 (23) | - |
|  | >5 a week |  | 132 (2) | - | - | 105 (2) | - |
| Sexual frequency at 18 years postpartum | Not at all | 3,788 (29) | 743 (20) | - | - | - | 608 (19) |
|  | < once a month |  | 669 (18) | - | - | - | 569 (18) |
|  | 1-3 times a month |  | 822 (22) | - | - | - | 689 (22) |
|  | About once a week |  | 821 (22) | - | - | - | 685 (22) |
|  | 2-4 times a week |  | 681 (18) | - | - | - | 579 (18) |
|  | >5 a week |  | 52 (1) | - | - | - | 39 (1) |

^a^ By category out of total number of participants with data for each characteristic

^b^ Crown Crisp Experimental Index (CCEI) for anxiety: threshold (≥8 for probable anxiety) as determined by authors Glover *et al*. (2004) ^1^

^c^ Edinburgh Postnatal Depression Scale (EPDS) for depression: threshold (≥13 for probable depression of varying degrees of severity) as determined by authors Cox *et al*. (1987) ^2^ of the 10-item EPDS

**Table S4.** Summary of the variables in the analysis model for both sex-related pain outcomes at 11 years postpartum, including the amount of data available for each variable in both the full dataset (after exclusions) and restricted to those with observed outcome data.

|  |  |  |  | **Pain the vagina during sex** | **Pain elsewhere after sex** |
| --- | --- | --- | --- | --- | --- |
| **Characteristic** | **Categories** | **Available data**  **(*n*=13,296)**  ***n* (%)** | **Available data by category^a^**  ***n* (% of available data)** | **Complete records 11 years**  **(*n*=5,384)**  ***n* (%)** | **Complete records 11 years**  **(*n*=5,372)**  ***n* (%)** |
| Mode of delivery | Vaginal delivery | 13,296 (100) | 11,845 (89) | 4,820 (90) | 4,809 (90) |
|  | Caesarean section |  | 1,451 (11) | 564 (10) | 563 (10) |
| Body mass index (BMI) | Underweight | 11,024 (83) | 553 (5) | 232 (4) | 233 (4) |
|  | Normal |  | 8,186 (74) | 4,082 (76) | 4,072 (76) |
|  | Overweight |  | 1,670 (15) | 796 (15) | 792 (15) |
|  | Obese |  | 615 (6) | 274 (5) | 275 (5) |
| Age | Under 25 | 13,296 (100) | 3,211 (24) | 777 (14) | 771 (14) |
|  | 25 and over |  | 10,085 (76) | 4,607 (86) | 4,601 (86) |
| Diabetes during pregnancy | No | 13,296  (100) | 13,190 (99) | 5,341 (99) | 5,328 (99) |
|  | Yes |  | 106 (1) | 43 (1) | 44 (1) |
| Educational attainment | A levels or less | 11,889 (89) | 10,374 (87) | 4,501 (84) | 4,488 (84) |
|  | University degree |  | 1,515 (13) | 883 (16) | 884 (16) |
| Anxiety | CCEI^b^ Score <8 | 11,241 (85) | 9,373 (83) | 4,650 (86) | 4,639 (86) |
|  | CCEI^b^ Score ≥8 |  | 1,868 (17) | 734 (14) | 733 (14) |
| Depression | EPDS^c^ Score <13 | 11,431 (86) | 9,841 (86) | 4,822 (90) | 4,812 (90) |
|  | EPDS^c^ Score ≥13 |  | 1,590 (14) | 562 (10) | 560 (10) |
| Parity | Nulliparous | 12,327 (93) | 5,485 (45) | 2,499 (46) | 2,496 (46) |
|  | Multiparous |  | 6,842 (55) | 2,885 (54) | 2,876 (54) |
| Pain in the vagina during sex at 11 years postpartum | Not at all | 6,611 (50) | 5,185 (78) | 4,213 (78) | - |
|  | Not a lot |  | 1,245 (19) | 1,017 (19) | - |
|  | Moderate |  | 133 (2) | 113 (2) | - |
|  | A lot |  | 48 (1) | 41 (1) | - |
| Pain elsewhere after sex at 11 years postpartum | Don’t have sex | 6,595 (50) | 173 (3) | - | 135 (3) |
|  | Never |  | 5,865 (89) | - | 4,807 (89) |
|  | Occasionally |  | 472 (7) | - | 361 (7) |
|  | Often |  | 69 (1) | - | 59 (1) |
|  | Always |  | 16 (0.2) | - | 10 (0.2) |

^a^ By category out of total number of participants with data for each characteristic

^b^ Crown Crisp Experimental Index (CCEI) for anxiety: threshold (≥8 for probable anxiety) as determined by authors Glover *et al*. (2004) ^1^

^c^ Edinburgh Postnatal Depression Scale (EPDS) for depression: threshold (≥13 for probable depression of varying degrees of severity) as determined by authors Cox *et al*. (1987) ^2^ of the 10-item EPDS

**Table S5.** Predictors of being a complete case in the full ALSPAC cohort (in those with complete exposure data *n*=13,299)^a^ from univariate models. No evidence of associations between observed sexual outcomes or mode of delivery and being a complete case (i.e. having data on all of exposures, confounders and outcomes); however some covariates were associated with missingness including BMI, age, educational attainment and mental health.

| **Characteristic** | **Category** | **Unadjusted OR**  **(95% CI)** |
| --- | --- | --- |
| Mode of delivery | Vaginal delivery | 1.00 (reference) |
|  | Caesarean section | 0.89 (0.79–1.00) |
| Body mass index (BMI) | Underweight | 1.00 (reference) |
|  | Normal | 1.26 (1.02–1.55) |
|  | Overweight | 1.40 (1.10–1.78) |
|  | Obese | 1.30 (0.97–1.73) |
| Age at delivery | Under 25 | 1.00 (reference) |
|  | 25 and over | 1.42 (1.32–1.54) |
| Maternal education | A levels or less | 1.00 (reference) |
|  | University degree | 1.28 (1.12–1.46) |
| Anxiety | CCEI^b^ Score <8 | 1.00 (reference) |
|  | CCEI^b^ Score ≥8 | 0.76 (0.67–0.85) |
| Depression | EPDS^c^ Score <13 | 1.00 (reference) |
|  | EPDS^c^ Score ≥13 | 0.61 (0.54–0.69) |
| Parity | Nulliparous | 1.00 (reference) |
|  | Multiparous | 1.00 (0.92–1.08) |
| **Sexual enjoyment** | | |
| Sexual enjoyment at 33 months postpartum | No sex at the moment | 1.00 (reference) |
|  | No, not at all | 1.01 (0.55–1.84) |
|  | No, not a lot | 1.15 (0.89–1.47) |
|  | Yes, somewhat | 1.13 (0.92–1.38) |
|  | Yes, a lot | 1.08 (0.88–1.31) |
| Sexual enjoyment at 5 years postpartum | No sex at the moment | 1.00 (reference) |
|  | No, not at all | 1.57 (0.78–3.17) |
|  | No, not a lot | 1.15 (0.87–1.51) |
|  | Yes, somewhat | 1.21 (0.98 to 1.49) |
|  | Yes, a lot | 1.13 (0.92–1.39) |
| Sexual enjoyment at 12 years postpartum | No sex at the moment | 1.00 (reference) |
|  | No, not at all | 1.18 (0.55–2.53) |
|  | No, not a lot | 1.15 (0.85–1.54) |
|  | Yes, somewhat | 1.30 (1.05–1.59) |
|  | Yes, a lot | 1.12 (0.92–1.37) |
| Sexual enjoyment at 18 years postpartum | No sex at the moment | 1.00 (reference) |
|  | No, not at all | 1.44 (0.59–3.48) |
|  | No, not a lot | 1.36 (0.92–2.01) |
|  | Yes, somewhat | 1.31 (1.01–1.69) |
|  | Yes, a lot | 1.04 (0.81–1.33) |
| **Sexual frequency** | | |
| Sexual frequency at 33 months postpartum | Not at all | 1.00 (reference) |
|  | < once a week | 1.13 (0.91–1.40) |
|  | 1 – 3 times a month | 1.26 (1.05–1.51) |
|  | About once a week | 1.11 (0.92–1.33) |
|  | 2-4 times a week | 1.01 (0.84–1.21) |
|  | > 5 times a week | 0.83 (0.58–1.17) |
| Sexual frequency at 5 years postpartum | Not at all | 1.00 (reference) |
|  | < once a week | 1.23 (0.98–1.56) |
|  | 1 – 3 times a month | 1.27 (1.05–1.54) |
|  | About once a week | 1.21 (1.00–1.47) |
|  | 2-4 times a week | 1.14 (0.94–1.38) |
|  | > 5 times a week | 0.92 (0.64–1.32) |
| Sexual frequency at 12 years postpartum | Not at all | 1.00 (reference) |
|  | < once a week | 1.42 (1.14–1.77) |
|  | 1 – 3 times a month | 1.46 (1.20–1.76) |
|  | About once a week | 1.38 (1.14–1.68) |
|  | 2-4 times a week | 1.31 (1.08–1.59) |
|  | > 5 times a week | 1.10 (0.73–1.66) |
| Sexual frequency at 18 years postpartum | Not at all | 1.00 (reference) |
|  | < once a week | 1.21 (0.92–1.59) |
|  | 1 – 3 times a month | 1.12 (0.87–1.45) |
|  | About once a week | 1.09 (0.84–1.40) |
|  | 2-4 times a week | 1.21 (0.92–1.59) |
|  | > 5 times a week | 0.66 (0.35–1.27) |
| **Pain outcomes** | | |
| Pain in the vagina during sex at 11 years postpartum | Not at all | 1.00 (reference) |
|  | Not a lot | 1.02 (0.89–1.18) |
|  | Moderate | 1.37 (0.89–2.12) |
|  | A lot | 1.39 (0.67–2.85) |
| Pain elsewhere after sex at 11 years postpartum | Don’t have sex | 1.00 (reference) |
|  | Never | 1.15 (0.82–1.62) |
|  | Occasionally | 0.86 (0.58–1.27) |
|  | Often | 1.34 (0.69–2.64) |
|  | Always | 0.35 (0.14–0.93) |

^a^ Each analysis in this Table has a different denominator depending on variable, questionnaire, and completion rate

^b^ Crown Crisp Experimental Index (CCEI) for anxiety: threshold (≥8 for probable anxiety) as determined by authors Glover *et al*. (2004) ^1^

^c^ Edinburgh Postnatal Depression Scale (EPDS) for depression: threshold (≥13 for probable depression of varying degrees of severity) as determined by authors Cox *et al*. (1987) ^2^ of the 10-item EPDS

**Table S6**. Summary of unobserved outcome data (out of the total with exposure data and at least one complete outcome), by those who had returned the questionnaire but selectively not answered the outcome-related question and those who did not return the entire questionnaire.

| **Outcome** | **Participants with missing outcome data**  ***n*** | **Participants with missing outcome data who completed the questionnaire**  ***n* (%)** | **Participants with missing outcome data who did not complete the questionnaire**  ***n* (%)** |
| --- | --- | --- | --- |
| Sexual enjoyment at 33 months postpartum | 1,287 | 219 (17.0) | 1,068 (83.0) |
| Sexual enjoyment at 5 years postpartum | 2,046 | 338 (16.5) | 1,708 (83.5) |
| Sexual enjoyment at 12 years postpartum | 4,246 | 418 (9.8) | 3,828 (90.2) |
| Sexual frequency at 18 years postpartum | 6,610 | 219 (3.3) | 6,319 (96.7) |
| Sexual frequency at 33 months postpartum | 1,186 | 118 (10.0) | 1,068 (90.0) |
| Sexual frequency at 5 years postpartum | 1,956 | 248 (12.7) | 1,708 (87.3) |
| Sexual frequency at 12 years postpartum | 4,117 | 289 (7.0) | 3,828 (93.0) |
| Sexual frequency at 18 years postpartum | 6,536 | 217 (3.3) | 6,319 (96.7) |
| Pain in the vagina during sex at 11 years postpartum | 3,713 | 383 (10.3) | 3,330 (89.7) |
| Pain elsewhere after sex at 11 years postpartum | 3,729 | 399 (10.7) | 3,330 (89.3) |

**Table S7**. In order to examine the potential that those who had selectively not answered outcome-related questions having returned the questionnaire might be missing not at random, proportion of missing was investigated for the previous question (prior to sexual-related questions) for each questionnaire and a randomly selected subsequent question. Proportions of missing in other questions amongst those who had returned each questionnaire would elucidate whether missingness might be due to chance, fatigue, or the true value of the outcome.

| **Timepoint**  ***n* (participants who completed questionnaire)** | **Missing in previous question**  ***n* (% out of total who completed questionnaire)** | **Missing in sexual frequency**  ***n* (% out of total who completed questionnaire)** | **Missing in sexual enjoyment**  ***n* (% out of total who completed questionnaire)** | **Missing in pain in the vagina during sex**  ***n* (% out of total who completed questionnaire)** | **Missing in pain elsewhere after sex**  ***n* (% out of total who completed questionnaire)** | **Missing in random question**  ***n* (% out of total who completed questionnaire)** |
| --- | --- | --- | --- | --- | --- | --- |
| **33 months** (9,256) | 79 (0.9) | 118 (1.3) | 219 (2.4) | - | - | 79 (0.9) |
| **5 years** (8,616) | 87 (1.0) | 248 (2.9) | 338 (3.9) | - | - | 86 (1.0) |
| **11 years** (6,994) | 37 (0.5) | - | - | 383 (5.5) | 399 (5.7) | 41 (0.6) |
| **12 years** (6,496) | 115 (1.8) | 289 (4.4) | 418 (6.4) | - | - | 101 (1.6) |
| **18 years** (4,005) | 109 (2.7) | 217 (5.4) | 291 (7.3) | - | - | 205 (5.1) |

| Odds ratio  (95% confidence interval) | **Sexual enjoyment at 33 months** | **Sexual enjoyment at 18 years** | **Sexual frequency at 33 months** | **Sexual frequency at 18 years** | **Pain in the vagina during sex** | **Pain elsewhere after sex** |
| --- | --- | --- | --- | --- | --- | --- |
| **Sexual enjoyment at 33 months** | - | 1.64  (1.53–1.74) | 3.50  (3.34–3.66) | 1.41  (1.33–1.50) | 0.89  (0.84–0.94) | 1.21  (1.11–1.31) |
| **Sexual enjoyment at 18 years** | 1.45  (1.39–1.53) | - | 1.31  (1.26–1.37) | 4.11  (3.84–4.39) | 0.86  (0.81–0.91) | 1.33  (1.22–1.46) |
| **Sexual frequency at 33 months** | 2.40  (2.31–2.49) | 1.31  (1.25–1.38) | - | 1.75  (1.67–1.84) | 0.92  (0.88–0.96) | 1.18  (1.11–1.26) |
| **Sexual frequency at 18 years** | 1.28  (1.23–1.34) | 2.90  (2.74–3.08) | 1.67  (1.60–1.75) | - | 0.93  (0.88–0.98) | 1.22  (1.13–1.32) |
| **Pain in the vagina during sex** | 0.72  (0.65–0.78) | 0.68  (0.61–0.77) | 0.84  (0.77–0.91) | 0.86  (0.77–0.97) | - | 2.96  (2.61–3.36) |
| **Pain elsewhere after sex** | 1.08  (0.95–1.22) | 1.40  (1.18–1.66) | 1.27  (1.13–1.44) | 1.45  (1.24–1.70) | 3.39  (2.95–3.91) | - |

**Table S8.** Associations from univariate models between each outcome variable and all other outcome variables to provide justification for using observed outcomes as auxiliaries for unobserved outcomes.

**B.2 – Primary analysis**

**Table S9.** Unadjusted and adjusted odds ratios (OR and aOR, respectively) for each outcome at each timepoint for the primary analysis in the imputed dataset (*n*=10,324) (Figure 3 in the main text): caesarean section compared with vaginal delivery.

|  | **OR**  **(95% CI)** | **P-value** | **aOR^a^**  **(95% CI)** | **P-value** |
| --- | --- | --- | --- | --- |
| **Sexual enjoyment** | - | - | - | - |
| 33 months | 1.06  (0.93–1.20) | 0.404 | 1.11  (0.97–1.27) | 0.116 |
| 5 years | 1.00  (0.88–1.15) | 0.948 | 1.07  (0.93–1.22) | 0.350 |
| 12 years | 1.04  (0.89–1.21) | 0.646 | 1.07  (0.92–1.26) | 0.380 |
| 18 years | 1.11  (0.91–1.35) | 0.315 | 1.21  (0.98–1.48) | 0.072 |
| **Sexual frequency** | - | - | - | - |
| 33 months | 0.87  (0.78–0.98) | 0.021 | 0.99  (0.88–1.12) | 0.844 |
| 5 years | 0.80  (0.71–0.90) | <0.001 | 0.92  (0.82–1.04) | 0.205 |
| 12 years | 0.90  (0.78–1.03) | 0.119 | 1.01  (0.88–1.16) | 0.897 |
| 18 years | 0.93  (0.79–1.10) | 0.420 | 1.09  (0.92–1.30) | 0.317 |
| **Pain in the vagina during sex** | - | - | - | - |
| 11 years | 1.68  (1.42–2.00) | <0.001 | 1.74  (1.46–2.08) | <0.001 |
| **Pain elsewhere after sex** | - | - | - | - |
| 11 years | 1.31  (1.01–1.68) | 0.038 | 1.43  (1.10–1.86) | 0.008 |

^a^ Adjusted for maternal age at delivery, maternal BMI at 12 weeks’ gestation, any diabetes during pregnancy, parity, anxiety and depression at 18 weeks’ gestation and maternal education at 32 weeks’ gestation

**B.3 – Additional analyses**

**B.3.1 – Instrumental vs non-instrumental vaginal delivery**

**Table S10**. Unadjusted and adjusted odds ratios (OR and aOR, respectively) for each outcome at each timepoint for the stratified vaginal delivery sensitivity analysis in the imputed dataset (*n*=9,230): instrumental vaginal delivery (forceps and vacuum deliveries) compared with non-instrumental vaginal deliveries (spontaneous vaginal and breech deliveries)

|  | **OR**  **(95% CI)** | **P-value** | **aOR^a^**  **(95% CI)** | **P-value** |
| --- | --- | --- | --- | --- |
| **Sexual enjoyment** | - | - | - | - |
| 33 months | 0.89  (0.79–1.01) | 0.079 | 0.91  (0.79–1.03) | 0.138 |
| 5 years | 0.97  (0.85–1.11) | 0.632 | 1.00  (0.87–1.15) | 0.990 |
| 12 years | 0.94  (0.81–1.09) | 0.402 | 0.95  (0.81–1.12) | 0.530 |
| 18 years | 0.92  (0.76–1.12) | 0.431 | 0.97  (0.79–1.19) | 0.757 |
| **Sexual frequency** | - | - | - | - |
| 33 months | 0.77  (0.68–0.86) | <0.001 | 0.92  (0.82–1.03) | 0.159 |
| 5 years | 0.79  (0.70–0.89) | <0.001 | 0.91  (0.81–1.03) | 0.147 |
| 12 years | 0.86  (0.75–0.98) | 0.020 | 0.88  (0.77–1.01) | 0.063 |
| 18 years | 0.91  (0.78–1.07) | 0.253 | 0.95  (0.81–1.13) | 0.578 |
| **Pain in the vagina during sex** | - | - | - | - |
| 11 years | 1.08  (0.90–1.28) | 0.415 | 1.05  (0.87–1.26) | 0.624 |
| **Pain elsewhere after sex** | - | - | - | - |
| 11 years | 0.97  (0.73–1.29) | 0.842 | 1.04  (0.77–1.41) | 0.781 |

^a^ Adjusted for maternal age at delivery, maternal BMI at 12 weeks’ gestation, any diabetes during pregnancy, parity, anxiety and depression at 18 weeks’ gestation and maternal education at 32 weeks’ gestation

**B.3.2 – Emergency C-section and elective C-section vs vaginal delivery**

**Table S11**. Unadjusted and adjusted odds ratios (OR and aOR, respectively) for each outcome at each timepoint for the stratified caesarean section sensitivity analysis in the imputed dataset (*n*=10,322): emergency and elective caesarean section (C-section) compared with vaginal delivery.

|  | **Exposure group** | **OR (95% CI)** | **P-value** | **aOR^a^ (95% CI)** | **P-value** |
| --- | --- | --- | --- | --- | --- |
| **Sexual enjoyment** |  | - | - | - | - |
|  | Vaginal delivery | 1.00 (reference) | - | 1.00 (reference) | - |
| 33 months | Emergency C-section | 1.04  (0.88–1.23) | 0.632 | 1.09  (0.92–1.29) | 0.312 |
|  | Elective C-section | 1.08  (0.88–1.33) | 0.437 | 1.15  (0.94–1.42) | 0.180 |
|  | Vaginal delivery | 1.00 (reference) | - | 1.00 (reference) | - |
| 5 years | Emergency C-section | 1.03  (0.87–1.21) | 0.752 | 1.09  (0.92–1.29) | 0.328 |
|  | Elective C-section | 0.96  (0.78–1.19) | 0.729 | 1.03  (0.83–1.27) | 0.781 |
|  | Vaginal delivery | 1.00 (reference) | - | 1.00 (reference) | - |
| 12 years | Emergency C-section | 1.03  (0.86–1.24) | 0.745 | 1.06  (0.88–1.28) | 0.549 |
|  | Elective C-section | 1.04  (0.83–1.32) | 0.721 | 1.09  (0.87–1.38) | 0.452 |
|  | Vaginal delivery | 1.00 (reference) | - | 1.00 (reference) | - |
| 18 years | Emergency C-section | 1.12  (0.90–1.39) | 0.320 | 1.20  (0.96–1.50) | 0.114 |
|  | Elective C-section | 1.09  (0.82–1.44) | 0.546 | 1.22  (0.92–1.62) | 0.171 |
| **Sexual frequency** |  | - | - | - | - |
|  | Vaginal delivery | 1.00 (reference) | - | 1.00 (reference) | - |
| 33 months | Emergency C-section | 0.86  (0.74–0.99) | 0.042 | 1.00  (0.86–1.16) | 0.968 |
|  | Elective C-section | 0.90  (0.75–1.08) | 0.249 | 0.99  (0.82–1.19) | 0.876 |
|  | Vaginal delivery | 1.00 (reference) | - | 1.00 (reference) | - |
| 5 years | Emergency C-section | 0.81  (0.70–0.94) | 0.006 | 0.95  (0.82–1.10) | 0.478 |
|  | Elective C-section | 0.78  (0.65–0.94) | 0.009 | 0.89  (0.73–1.07) | 0.221 |
|  | Vaginal delivery | 1.00 (reference) | - | 1.00 (reference) | - |
| 12 years | Emergency C-section | 0.91  (0.77–1.06) | 0.230 | 1.00  (0.85–1.18) | 0.992 |
|  | Elective C-section | 0.89  (0.72–1.09) | 0.252 | 1.03  (0.84–1.27) | 0.771 |
|  | Vaginal delivery | 1.00 (reference) | - | 1.00 (reference) | - |
| 18 years | Emergency C-section | 0.97  (0.80–1.17) | 0.733 | 1.11  (0.91–1.35) | 0.315 |
|  | Elective C-section | 0.89  (0.70–1.12) | 0.309 | 1.07  (0.84–1.37) | 0.568 |
| **Pain in the vagina during sex** |  | - | - | - | - |
|  | Vaginal delivery | 1.00 (reference) | - | 1.00 (reference) | - |
| 11 years | Emergency C-section | 1.67  (1.36–2.05) | <0.001 | 1.70  (1.37–2.10) | <0.001 |
|  | Elective C-section | 1.71  (1.33–2.20) | <0.001 | 1.83  (1.41–2.37) | <0.001 |
| **Pain elsewhere after sex** |  | - | - | - | - |
|  | Vaginal delivery | 1.00 (reference) | - | 1.00 (reference) | - |
| 11 years | Emergency C-section | 1.28  (0.94–1.73) | 0.116 | 1.39  (1.01–1.91) | 0.043 |
|  | Elective C-section | 1.35  (0.93–1.96) | 0.112 | 1.48  (1.01–2.18) | 0.044 |

^a^ Adjusted for maternal age at delivery, maternal BMI at 12 weeks’ gestation, any diabetes during pregnancy, parity, anxiety and depression at 18 weeks’ gestation and maternal education at 32 weeks’ gestation

**B.4 – Sensitivity analyses**

**B.4.1 – Worst case scenario**

**Table S12.** Unadjusted and adjusted odds ratios (OR and aOR, respectively) for each outcome at each timepoint for the worst case scenario sensitivity analysis in the sensitivity analysis imputed dataset (*n*=10,324): caesarean section compared with vaginal delivery.

|  | **OR**  **(95% CI)** | **P-value** | **aORa**  **(95% CI)** | **P-value** |
| --- | --- | --- | --- | --- |
| **Sexual enjoyment** | - | - | - | - |
| 33 months | 1.03  (0.91–1.16) | 0.646 | 1.10  (0.97–1.24) | 0.154 |
| 5 years | 0.94  (0.83–1.07) | 0.349 | 1.01  (0.89–1.15) | 0.905 |
| 12 years | 0.99  (0.87–1.14) | 0.926 | 1.06  (0.92–1.22) | 0.410 |
| 18 years | 1.03  (0.87–1.21) | 0.731 | 1.18  (0.99–1.40) | 0.058 |
| **Sexual frequency** | - | - | - | - |
| 33 months | 0.87  (0.77–0.98) | 0.017 | 0.99  (0.88–1.11) | 0.833 |
| 5 years | 0.78  (0.70–0.88) | <0.001 | 0.90  (0.80–1.01) | 0.083 |
| 12 years | 0.87  (0.76–0.99) | 0.038 | 0.97  (0.85–1.11) | 0.683 |
| 18 years | 0.92  (0.78–1.07) | 0.269 | 1.07  (0.91–1.26) | 0.413 |
| **Pain in the vagina during sex** | - | - | - | - |
| 11 years | 1.48  (1.27–1.73) | <0.001 | 1.51  (1.29–1.78) | <0.001 |
| **Pain elsewhere after sex** | - | - | - | - |
| 11 years | 1.18  (0.97–1.42) | 0.090 | 1.21  (1.00–1.48) | 0.051 |

^1^ Adjusted for maternal age at delivery, maternal BMI at 12 weeks’ gestation, any diabetes during pregnancy, parity, anxiety and depression at 18 weeks’ gestation and maternal education at 32 weeks’ gestation

**B.4.2 – Additional adjustment for health problems**

**Table S13**. Unadjusted and adjusted odds ratios (OR and aOR, respectively) for each outcome at each timepoint for mode of delivery in the imputed dataset (*n*=10,324): caesarean section vs vaginal delivery.

|  | **OR**  **(95% CI)** | **P-value** | **aOR^a^**  **(95% CI)** | **P-value** |
| --- | --- | --- | --- | --- |
| **Sexual enjoyment** | - | - | - | - |
| 33 months | 1.06  (0.93–1.20) | 0.408 | 1.12  (0.98–1.28) | 0.102 |
| 5 years | 1.00  (0.88–1.15) | 0.973 | 1.07  (0.93–1.23) | 0.331 |
| 12 years | 1.04  (0.88–1.22) | 0.635 | 1.08  (0.91–1.28) | 0.362 |
| 18 years | 1.10  (0.90–1.34) | 0.367 | 1.20  (0.98–1.48) | 0.082 |
| **Sexual frequency** | - | - | - | - |
| 33 months | 0.87  (0.77–0.98) | 0.020 | 0.99  (0.88–1.12) | 0.872 |
| 5 years | 0.80  (0.71–0.90) | <0.001 | 0.93  (0.82–1.05) | 0.220 |
| 12 years | 0.89  (0.78–1.02) | 0.082 | 1.00  (0.87–1.14) | 0.995 |
| 18 years | 0.94  (0.80–1.10) | 0.445 | 1.11  (0.94–1.30) | 0.237 |
| **Pain in the vagina during sex** | - | - | - | - |
| 11 years | 1.65  (1.38–1.96) | <0.001 | 1.68  (1.40–2.02) | <0.001 |
| **Pain elsewhere after sex** | - | - | - | - |
| 11 years | 1.29  (0.99–1.68) | 0.061 | 1.38  (1.05–1.83) | 0.022 |

^a^ Adjusted for maternal age at delivery, maternal BMI at 12 weeks’ gestation, any diabetes during pregnancy, parity, anxiety and depression at 18 weeks’ gestation and general health and maternal education at 32 weeks’ gestation

**B.4.2 – Complete case analysis**

**Table S14.** Unadjusted and adjusted odds ratios (OR and aOR, respectively) for each outcome at each timepoint for the complete case analysis: caesarean section compared with vaginal delivery.

|  | **Caesarean section** | **Vaginal delivery** | **OR**  **(95% CI)** | **P-value** | **aOR^a^**  **(95% CI)** | **P-value** |
| --- | --- | --- | --- | --- | --- | --- |
| **Sexual enjoyment** | - | - | - | - | - | - |
| 33 months | 680 | 6,017 | 1.09  (0.94–1.27) | 0.265 | 1.15  (0.99–1.35) | 0.077 |
| 5 years | 633 | 5,536 | 1.03  (0.88–1.21) | 0.734 | 1.10  (0.93–1.29) | 0.258 |
| 12 years | 465 | 4,044 | 1.06  (0.88–1.28) | 0.527 | 1.11  (0.91–1.34) | 0.303 |
| 18 years | 268 | 2,382 | 1.13  (0.88–1.39) | 0.483 | 1.13  (0.88–1.44) | 0.344 |
| **Sexual frequency** | - | - | - | - | - | - |
| 33 months | 740 | 6,514 | 0.86  (0.76–0.99) | 0.035 | 0.99  (0.86–1.13) | 0.853 |
| 5 years | 684 | 5,991 | 0.82  (0.72–0.94) | 0.005 | 0.96  (0.83–1.10) | 0.530 |
| 12 years | 514 | 4,545 | 0.94  (0.80–1.10) | 0.444 | 1.06  (0.90–1.24) | 0.518 |
| 18 years | 316 | 2,853 | 0.96  (0.78–1.18) | 0.688 | 1.14  (0.92–1.40) | 0.233 |
| **Pain in the vagina during sex** | - | - | - | - | - | - |
| 11 years | 564 | 4,820 | 1.72  (1.42–2.08) | <0.001 | 1.82  (1.49–2.21) | <0.001 |
| **Pain elsewhere after sex** | - | - | - | - | - | - |
| 11 years | 547 | 4,690 | 1.50  (1.13–1.99) | 0.005 | 1.70  (1.27–2.28) | <0.001 |

^a^ Adjusted for maternal age at delivery, maternal BMI at 12 weeks’ gestation, any diabetes during pregnancy parity, anxiety and depression at 18 weeks’ gestation and maternal education at 32 weeks’ gestation

**B.4.3 – Complete case analysis in nulliparous women**

**Table S15.** Unadjusted and adjusted odds ratios (OR and aOR, respectively) for each outcome at each timepoint for the complete case analysis in nulliparous women: caesarean section compared with vaginal delivery.

|  | **Caesarean section** | **Vaginal delivery** | **OR**  **(95% CI)** | **P-value** | **aOR^a^**  **(95% CI)** | **P-value** |
| --- | --- | --- | --- | --- | --- | --- |
| **Sexual enjoyment** | - | - | - | - | - | - |
| 33 months | 362 | 2,601 | 1.05  (0.85–1.30) | 0.629 | 1.19  (0.95–1.47) | 0.126 |
| 5 years | 336 | 2,434 | 1.02  (0.82–1.28) | 0.828 | 1.17  (0.93–1.47) | 0.189 |
| 12 years | 261 | 1,861 | 1.13  (0.88–1.45) | 0.341 | 1.22  (0.94–1.58) | 0.130 |
| 18 years | 150 | 1,102 | 0.97  (0.70–1.34) | 0.862 | 1.08  (0.77–1.51) | 0.670 |
| **Sexual frequency** | - | - | - | - | - | - |
| 33 months | 402 | 2,862 | 0.91  (0.75–1.09) | 0.301 | 1.06  (0.87–1.28) | 0.576 |
| 5 years | 371 | 2,649 | 0.84  (0.69–1.01) | 0.065 | 0.99  (0.81–1.20) | 0.917 |
| 12 years | 293 | 2,095 | 0.89  (0.71–1.10) | 0.276 | 1.06  (0.85–1.33) | 0.610 |
| 18 years | 176 | 1,324 | 0.85  (0.65–1.12) | 0.253 | 1.05  (0.79–1.39) | 0.747 |
| **Pain in the vagina during sex** | - | - | - | - | - | - |
| 11 years | 318 | 2,181 | 1.61  (1.24–2.08) | <0.001 | 1.61  (1.23–2.10) | 0.001 |
| **Pain elsewhere after sex** | - | - | - | - | - | - |
| 11 years | 309 | 2,122 | 1.11  (0.73–1.70) | 0.622 | 1.29  (0.83–2.01) | 0.253 |

^a^ Adjusted for maternal age at delivery, maternal BMI at 12 weeks’ gestation, any diabetes during pregnancy, parity, anxiety and depression at 18 weeks’ gestation and maternal education at 32 weeks’ gestation

**B.4.4 – Using a generalized ordered logit model for sexual frequency at 5 years**

**Table S16**. Unadjusted and adjusted generalized ordered logit models for sexual frequency at 5 years in the complete case analysis cohort to account for proportional odds assumption violation: caesarean section compared to vaginal delivery.

|  | **Caesarean section** | **Vaginal delivery** | **OR**  **(95% CI)** | **P-value** | **aOR^a^**  **(95% CI)** | **P-value** |
| --- | --- | --- | --- | --- | --- | --- |
| **Sexual frequency at 5 years postpartum** | - | - | - | - | - | - |
| Not at all | 684 | 5,991 | 1.09  (0.83–1.45) | 0.529 | 1.30  (0.98–1.73) | 0.070 |
| < once a month |  |  | 0.87  (0.72–1.05) | 0.150 | 1.02  (0.84–1.24 | 0.852 |
| 1–3 times a month |  |  | 0.86  (0.74–1.01) | 0.071 | 1.00  (0.85–1.18) | 0.985 |
| About once a week |  |  | 0.69  (0.57–0.83) | <0.001 | 0.80  (0.66–0.97) | 0.020 |
| 2–4 times a week |  |  | 0.81  (0.44–1.46) | 0.476 | 0.96  (0.53–1.74) | 0.881 |
| 5+ times a week |  |  | 1.00  (reference) | – | 1.00 (reference) | – |

^a^ Adjusted for maternal age at delivery, maternal BMI at 12 weeks’ gestation, any diabetes during pregnancy, parity, anxiety and depression at 18 weeks’ gestation and maternal education at 32 weeks’ gestation

The generalized ordered logit model suggested that those who delivered via caesarean section were more likely to report having sex 2–4 times a week than not at all, compared with women who gave birth vaginally.

**B.5 – Distribution of responses by exposure**

**Supplementary Figure 1**. Distribution of responses for sexual enjoyment at each timepoint and pain in the vagina during sex at 11 years postpartum by vaginal delivery and caesarean section.

VD = vaginal delivery, CS = caesarean section

**Supplementary Figure 2**. Distribution of responses for sexual frequency at each timepoint and pain elsewhere after sex at 11 years postpartum by vaginal delivery and caesarean section.

VD = vaginal delivery, CS = caesarean section

**C – References**

1. Glover V, O'Connor TG, Heron J, Golding J, team AS. Antenatal maternal anxiety is linked with atypical handedness in the child. Early Hum Dev. 2004;79(2):107-18.

2. Cox JL, Holden JM, Sagovsky R. Detection of postnatal depression. Development of the 10-item Edinburgh Postnatal Depression Scale. Br J Psychiatry. 1987;150:782-6.

3. Matijasevich A, Victora CG, Lawlor DA, Golding J, Menezes AM, Araujo CL, et al. Association of socioeconomic position with maternal pregnancy and infant health outcomes in birth cohort studies from Brazil and the UK. J Epidemiol Community Health. 2012;66(2):127-35.

4. Lee KJ, Tilling KM, Cornish RP, Little RJA, Bell ML, Goetghebeur E, et al. Framework for the treatment and reporting of missing data in observational studies: The Treatment And Reporting of Missing data in Observational Studies framework. J Clin Epidemiol. 2021;134:79-88.
