# Supplementary figures and images for: Mode of delivery and maternal sexual wellbeing: A longitudinal study
Source: BJOG. 2022 Aug 3;129(12):2010–8. doi: 10.1111/1471-0528.17262 (PMC9804306; doi:10.1111/1471-0528.17262)

## Sexual enjoyment

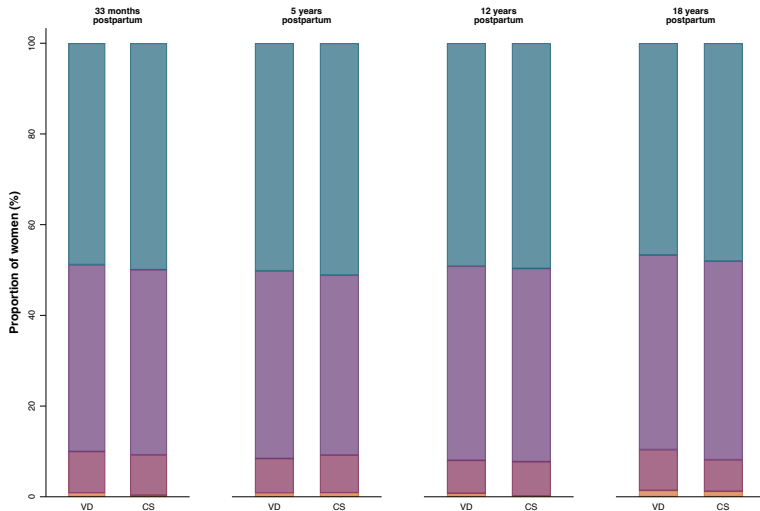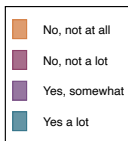

## Pain in the vagina during sex

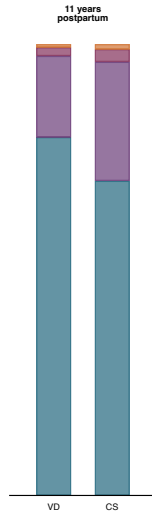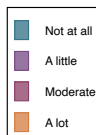

Supplement: Supplementary file 2 — Figure S1 [file BJO-129-2010-s008.pdf]

## Sexual frequency

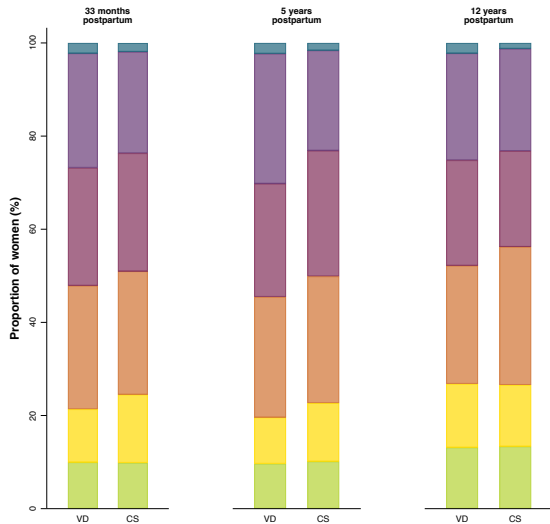

## Pain elsewhere after sex

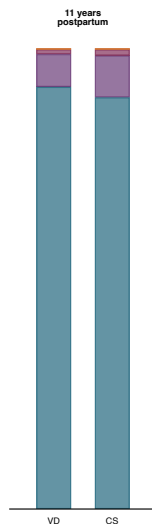

Supplement: Supplementary file 3 — Figure S2 [file BJO-129-2010-s002.pdf]
